# Medical elective research – eyelid asymmetry amongst Malaysians: a study of two racial groups

**DOI:** 10.1186/1753-6561-6-S4-P39

**Published:** 2012-07-09

**Authors:** WC Ngu, CN Chua

**Affiliations:** 1 University of Dundee, UK; 2 University Malaysia Sarawak, Malaysia

## Introduction

Facial asymmetry is a common condition and only becomes a problem if there is a significant difference between both sides. In the Asian community, the difference between the height and width of the palpebral fissures and the skin creases is usually the most apparent on photographs. There has not been any reports on the prevalence of such differences between the various racial groups in Malaysia. This study looks at the incidence of asymmetry of the palpebral fissue and upper eyelid crease in young Malaysians of Malay and Chinese origins of which form the biggest racial groups in Malaysia. Data obtained from such studies would be useful for reconstructive and aesthetic surgery as well as for counselling patients with dysmorphophobia.

## Methods

Malaysian students between the ages of 18 and 26 were randomly selected at University Malaysia Sarawak. These students comprised of 197 Malays and 198 Chinese. The following parameters of both eyes were obtained with a millimeter ruler: palpebral fissure width (PFW), palpebral fissure height (PFH), intercanthal distance (ICD) and upper eyelid skin crease height (UESC). The presence and absence of skin creases were also recorded. Data collected was analyzed with Microsoft Excel and SPSS.

## Results

There is statistical significant difference between the different sexes and races for PFW and PFH but none between the two sides. There is statistical significant difference between the two eyes for UESC. Chinese males have the highest percentage of PFW and PFH asymmetry. Malay males have the highest percentage of UESC asymmetry.

## Conclusions

Eyelid asymmetry is common amongst Malaysians. Asymmetry of PFW, PFH and UESC is commoner in males than females. Of the three parameters, asymmetry of UESC is the commonest. PFW and PFH asymmetry is more common in Chinese than Malays but UESC asymmetry is more common in Malays. UESC is almost universally present in Malays whereas in the Chinese there is a significant percentage of subjects with asymmetry of presence of UESC (9.52% in males and 9.46% in females) which gives rise to a more obvious eyelid asymmetry. This data is useful for oculoplastic surgeons who are keen on counselling patients who wish to undergo aesthetic procedures pre and post-operatively.

